# Extension and update of multiscale monthly household carbon footprint in Japan from 2011 to 2022

**DOI:** 10.1038/s41597-023-02329-2

**Published:** 2023-07-08

**Authors:** Liqiao Huang, Sebastian Montagna, Yi Wu, Zhiheng Chen, Kenji Tanaka, Yoshikuni Yoshida, Yin Long

**Affiliations:** 1 Graduate School of Engineering, University of Tokyo, Tokyo, Japan; 2 Bartlett School of Sustainable Construction, University College London, London, WC1E 7HB UK

**Keywords:** Environmental impact, Psychology and behaviour

## Abstract

Household consumption significantly contributes to greenhouse gas emissions as it is the largest component of final demand in the national accounting system. Nevertheless, there is an apparent lack of comprehensive and consistent datasets detailing emissions from household consumption. Here, we expand and update Japan’s multiscale monthly household carbon footprint from January 2011 to September 2022, combining data from government statistics and surveys. We constructed a dataset comprising 37,692 direct and 4,852,845 indirect emission records, covering households at the national, regional, and prefectural city levels. The dataset provides critical spatiotemporal information that allows for revealing carbon emission patterns, pinpointing primary sources of emissions, and discerning regional variances. Moreover, the inclusion of micro-scale carbon footprint data enables the identification of specific consumption habits, thereby regulating individual consumption behavior to achieve a low-carbon society.

## Background & Summary

Climate change and increasing greenhouse gas (GHG) emissions present a formidable challenge in contemporary times^1^, and the predominant driving force behind this is the accrual of GHG in the atmosphere, particularly carbon dioxide (CO_2_), which contributes to environmental alterations, including global warming^2,3^. Given the extensive ramifications of climate change, nations and institutions have instituted carbon neutrality objectives to achieve climate change mitigation goals^4–6^. Global emission reduction pathways, such as those outlined in the Paris Agreement and the Net Zero Pathways, propose specific strategies and benchmarks to achieve these objectives^7–9^. It is imperative to transition towards sustainable consumption and production paradigms that foster resource efficiency, waste minimization, pollution abatement, and ecologically viable production processes to achieve these goals^10–12^. Such goals are also congruent with multiple sustainable development goals (SDGs), encompassing Sustainable Cities and Communities (SDG 11), Responsible Consumption and Production (SDG 12), and Climate Action (SDG 13)^13^.

Although multiple parties have set targets and commitments, achieving carbon reduction targets is complex and challenging, requiring a comprehensive assessment of emission sources and mitigation potential. To achieve this, multiscale quantification is considered an essential instrument for assessing the progress made in the pursuit of sustainable development^14–16^. The carbon footprint has been quantified across various scales, including global^17,18^, national^19–22^, and city levels^23–26^. Recently, subnational carbon emission reduction pathways have garnered increasing interest as they acknowledge the significance of implementing tailored mitigation strategies suitable for specific circumstances^26–28^ and complement national-level policies. Engaging local communities in the development and implementation of mitigation strategies can facilitate greater participation of local stakeholders in the decision-making process^29,30^. Particularly, urban areas, contributing to approximately 70% of global carbon emissions^31,32^, play a vital role in mitigating the impacts of climate change and actualizing the SDGs^33–35^. Therefore, it is critical to assess the carbon footprint at the city level and develop carbon reduction strategies that identify specific emission sources and leverage local resources and capacities, including urban planning, transportation, energy systems, and waste management. By implementing these targeted measures at the city level, co-benefits, such as improving air quality, enhancing energy security, and creating new economic opportunities, can also be promoted^36,37^.

Within the urban context, it is essential to identify specific components within cities that should assume responsibility for implementing climate-change mitigation policies to refine the focus on the significance of urban areas^38–40^. According to various previous demonstrations and analyses, household consumption is a vital component of urban emissions^41–43^, and the necessity of decarbonization has become increasingly critical owing to climate-related concerns. Consequently, understanding the nexus between household behavior and the environment is paramount, given that the production and provision of goods and services are chiefly oriented toward fulfilling the ultimate demand of households^19,44–46^. On this basis, the household sector concurrently holds a crucial bearing on achieving emission reduction objectives and has considerable mitigation potential^47,48^. Evidence has demonstrated that seemingly trivial day-to-day actions, such as reducing water usage, turning off lights when not in use, and proper waste disposal, carry weight^49–52^. Therefore, this dataset focuses on the carbon footprint of Japan’s households, aiming to contribute to the body of knowledge that guides emission reduction strategies from the household perspective.

In our previous research^41^, we analyzed monthly direct and indirect GHG emissions for 51–52 Japanese cities, spanning 2011 to 2015. The dataset encompasses 1,555,512 items, with 1,543,128 items for indirect emissions and 12,384 items for direct emissions, which are publicly accessible via Figshare in the form of 17 Excel files. However, a considerable research gap exists due to the limited scope of the previous dataset, which only extended to 2015 and cannot reflect the impact of the COVID-19 pandemic. Therefore, an updated version of the dataset has been produced and updated up to September 2022. Furthermore, since emission reduction policies at national, prefectural, and city levels are interconnected and can reinforce each other, quantifying household carbon footprints at all levels aids in targeted policy creation, enabling effective interventions and providing households with insights to encourage lower-carbon lifestyles. Given that, the latest iteration of our research offers expanded spatial coverage. This includes carbon footprints at the national level, regional level (comprising 10 regions), as well as the average of large, medium, and small cities. The regions include the Hokkaido, Tohoku, Kanto, Hokuriku, Tokai, Kinki, Chugoku, Shikoku, Kyushu, and Okinawa regions. The updated dataset incorporates direct emission data from the use of natural gas, gasoline, liquefied petroleum gas (LPG), and kerosene, along with 515 consumption items that contribute to indirect emissions.

## Methods

### Scope of the dataset

The dataset is based on the Family Income and Expenditure Survey (FIES), conducted monthly by the Statistics Bureau of Japan^53^. This survey consistently quantifies the expenditures of Japanese households in approximately 500 different categories of goods and services.

**Fig. 1:**
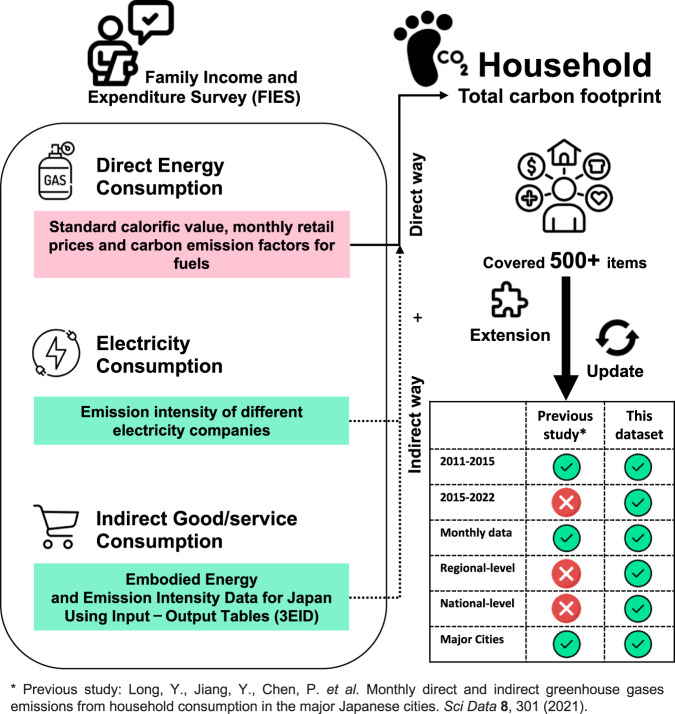
Flowchart of the dataset establishment.

The dataset described in this data descriptor includes direct and indirect monthly emissions from households at the national, regional, and city levels in Japan. Regarding the research period, household consumption data from January 2011 to September 2022 are covered. Compared with our previous research, this study adds the carbon footprint embodied in the 515 household consumption items for all months in the extended dataset, as elucidated in the Excel file labeled ‘Category.xlsx’. For direct emissions, the current dataset contains results from the use of four types of fuels. In addition to the national level, this study extracts consumption data for ‘Large cities,’ ‘Medium cities,’ ‘Small cities A,’ and ‘Small cities B/towns and villages’ to analyze the carbon footprint at these levels (regarding how to define large, medium, and small cities, please refer to the file titled ‘Classification of cities and other areas.xlsx’). In addition, we also provide 10 regional-specific household carbon footprints within the same time span. From the standpoint of cities, the discussion in 2011–2012 included 51 cities (primarily prefectural level cities), whereas household consumption data from 52 cities was used for analyzing the carbon footprint from 2013 to 2022, including the addition of Sagamihara. The process of establishing the dataset is illustrated in the flowchart in Fig. 1.

### Direct emission

A previous study identified gasoline, kerosene, LPG, and city gas as the primary fossil fuels responsible for direct emissions from Japanese households^41,54^. The fundamental principles for calculating the direct emissions from the use of different fuels are presented here. First, it involves the extraction of household expenditures from the FIES, followed by the conversion of consumption to mass or volume based on retail fuel prices, as Eq. (1) depicts:

1 $${P}_{i,j,m,y}^{{\rm{direct}}}={c}_{i,j,m,y}/\left(h{s}_{j,m,y}\cdot {u}_{i,j,m,y}\right)$$

where ${P}_{i,j,m,y}^{{\rm{direct}}}$ is the physical quantity of fuel type *i* in diverse space coverage *j* during month *m* of year *y*. Here, ‘diverse space coverage’ refers to the measurement of fuel quantity across various spatial scales, including national, regional and city scales (large, medium, small cities), as previously specified. Moreover, ${c}_{i,j,m,y}$ refers to the expenditure on fuel type *i* in diverse space coverage, and $h{s}_{j,m,y}$ indicates the average household size of diverse space coverage *j* in specific months, both captured from the FIES dataset. The term *u*_*i,j,m,y*_ refers to the retail price of fuel *i*, which is also related to the research area and period.

Next, the emission intensity, $E{F}_{i,j,m,y}^{{\rm{direct}}}$ of all four fuel types were estimated. Specifically, this computation relies on several factors, including the standard carbon emission coefficient ${s}_{i,j,m,y}$, the standard heat generation coefficient of fuels *h*_*i,m,y*_, and the conversion of carbon content in its chemical composition to CO_2_ equivalent, as shown in Eq. (2).

2 $$E{F}_{i,j,y}^{{\rm{direct}}}={s}_{i,j,m,y}\cdot {h}_{i,m,y}\cdot \left(\frac{{M}_{C{O}_{2}}}{{M}_{C}}\right)$$

Here, *h*_*i,m,y*_ is also updated monthly. Regarding the carbon content in the chemical composition, the corresponding CO_2_ equivalent is calculated by multiplying the carbon content by the ratio of the molar mass of CO_2_ (${M}_{C{O}_{2}}$, 44 g/mol) to the molar mass of carbon (*M*_*C*_, 12 g/mol).

Subsequently, multiplying the fuel mass or volume by the pertinent emission coefficients results in the computation of the monthly direct emissions, expressed in g-CO_2_e. The per capita direct emission ${E}_{i,j,m,y}^{{\rm{direct}}}$ is derived using Eq. (3),

3 $${E}_{i,j,m,y}^{{\rm{direct}}}={P}_{i,j,m,y}^{{\rm{direct}}}\cdot E{F}_{i,j,y}^{{\rm{direct}}}$$

where ${E}_{i,j,m,y}^{{\rm{direct}}}$ is the per capita direct emissions from the consumption of fuel type *i* in diverse space coverage *j* during month *m* of year *y*. The heating value of a fuel refers to the amount of heat released per unit mass (or unit volume) of the fuel upon complete combustion, that is, the standard carbon emission coefficient (based on total heat generation).

Notably, this study improves on the previous research by ensuring the reliability and availability of the data sources. The standard heat generation coefficient information is sourced from the ‘List of standard heat generation and carbon emission coefficients by energy source 2022’ published by the Ministry of the Environment, Japan^55^. Further details regarding the different fuels used are provided below.

For city gas, the data source is the FIES Monthly Prices and Annual Average Prices by Item dataset. This dataset is updated monthly and provides information on natural gas prices in different cities. The unit of the city gas price data is measured by the heat value, namely ‘for domestic use, early payment, 1465.12 MJ’. The carbon footprint of city gas can be calculated by comparing household spending on natural gas with the standard carbon emissions coefficient.

The data for kerosene and gasoline were obtained from a weekly survey of retail prices at filling stations conducted by the Ministry of Economy, Trade, and Industry of Japan^56^. This dataset is updated weekly and provides information on fuel prices in different counties. Unlike city gas data, this dataset provides fuel prices in yen per liter for gasoline and yen per 18 liters for kerosene. The carbon footprints of these fuels can be calculated by comparing household spending on gasoline and kerosene with their respective calorific values and standard carbon emission coefficients.

The price information for LPG was sourced from the Oil Information Center at the Institute of Energy Economics, Japan^57^. The retail price of LPG is based on consumption, with cutoffs of 5 m^3^, 10 m^3^, 20 m^3^, and 50 m^3^. Therefore, the average monthly household consumption of LPG in each region was determined by referencing the LPG consumption survey of Japan conducted by the Oil Information Center^58^.

### Indirect emission

To accurately assess the indirect carbon emissions associated with goods and services utilized by households, this study used the Embodied Energy and Emission Intensity Data for Japan Using Input-output Tables (3EID)^59^ and the FIES dataset. The FIES provides information on family income and expenditure, including consumption levels and sources of income and disparities in income and spending patterns across different income groups, presented in two volumes at the national and regional levels. Here, various GHGs, including CO_2_, CH_4_, N_2_O, HFCS, PFCS, SF_6_, and NF_3_, were considered and measured in CO_2_ equivalents, referred to as carbon footprints. According to the FIES dataset, the sample was updated at regular intervals to minimize potential bias in the obtained data and alleviate the burden of long-term bookkeeping for the sampled households.

The calculation principle in the 3EID database of indirect carbon emission intensity is summarized as follows: First, energy consumption and air pollutant emissions were analyzed from a sector and fuel-type perspective, with 400 sectors consolidated into 17 sectors, revealing direct energy consumption and emissions quantitatively. The contribution of each sector’s environmental efforts to the total burden was calculated based on the final economic demand. In the first step, Eq. (4) was used to determine the indirect intensity.

4 $${\left(\begin{array}{c}{I}_{1,t}^{{\rm{indirect}}}\\ \vdots \\ {I}_{j,t}^{{\rm{indirect}}}\\ \vdots \\ {I}_{n,t}^{{\rm{indirect}}}\end{array}\right)}^{T}={\bf{D}}{\left({\bf{I}}-({\bf{I}}-\bar{{\bf{M}}}){\bf{A}}\right)}^{-1}$$

Here, ${I}_{j,t}^{{\rm{indirect}}}$ refers to the indirect intensity in sector *j* in year *t*, ${\bf{D}}=\left[{d}_{1},{d}_{2},{d}_{3},\ldots ,{d}_{n}\right]$ represents the direct emission intensity 1 ×*n* vector, and **I** represents the unit matrix. Therefore, the left side of Eq. (4) shows a transposed 1 ×*n* vector marked with the superscript *T*. **A** is the output requirement coefficient matrix, which is calculated by dividing industry *i’*s output needed to produce industry j*’*s output *x*_*ij*_ by the total output of sector *X*_*j*_ $\left({\bf{A}}=\left[{A}_{ij}\right]=\left[\frac{{x}_{ij}}{{X}_{j}}\right]\right)$, and $\bar{{\bf{M}}}$ is the diagonal matrix that represents the direct requirement coefficients for the import portion.

Although the 3EID database provides indirect emission intensities for a wide range of household consumer products, it does not completely match the industry classifications and expenditure data covered by the FIES database and only provides indirect emission intensities for 2011 and 2015, as the 3EID database’s release cycle is every five years. Therefore, we first remapped the emissions intensity and consumption data categories to align the indirect emission intensities from the 3EID database with the industry classifications and expenditure data from the FIES database. It should be noted that the 3EID emission intensity dataset provided results only for 2011 and 2015, with 395 and 390 items, respectively. By cross-mapping the 3EID dataset with the corresponding FIES dataset, we generated an emission inventory of 495 items between 2011 and 2014, 512 items between 2015 and 2019, and 504 items between 2020 and 2022. Second, to bridge the indirect emissions intensity data for the missing years in the 3EID database, we combined the interpolation method with information on inflation and the Consumer Price Index (CPI). To note, in this study, consumer price is employed, which adjusts the commercial and transportation margin rates when compared with producer prices, to accurately account for indirect emissions in household consumption^60^. The estimation process for the embodied carbon emissions intensity ${I}_{j,m,y}^{{\rm{indirect}}}$ of item *j* in year *y* is expressed in Eq. (5).

5 $$\left\{\begin{array}{c}{I}_{j,2012}^{{\rm{indirect}}}=\frac{3}{4}{I}_{j,2011}^{{\rm{indire}}c{\rm{t}}}+\frac{1}{4}{I}_{j,2015}^{{\rm{indirect}}}\\ {I}_{j,2013}^{{\rm{indirect}}}=\frac{1}{2}{I}_{j,2011}^{{\rm{indirect}}}+\frac{1}{2}{I}_{j,2015}^{{\rm{indirect}}}\\ {I}_{j,2014}^{{\rm{indirect}}}=\frac{1}{4}{I}_{j,2011}^{{\rm{indirect}}}+\frac{3}{4}{I}_{j,2015}^{{\rm{indirect}}}\\ {I}_{j,2016}^{{\rm{indirect}}}=IN{F}_{j,2016}\ast {I}_{j,2015}^{{\rm{indirect}}}\\ {I}_{j,2017}^{{\rm{indirect}}}=IN{F}_{j,2017}\ast {I}_{j,2015}^{{\rm{indirect}}}\\ {I}_{j,2018}^{{\rm{indirect}}}=IN{F}_{j,2018}\ast {I}_{j,2015}^{{\rm{indirect}}}\\ {I}_{j,2019}^{{\rm{indirect}}}=IN{F}_{j,2019}\ast {I}_{j,2015}^{{\rm{indirect}}}\\ {I}_{j,2020}^{{\rm{indirect}}}=IN{F}_{j,2020}\ast {I}_{j,2015}^{{\rm{indirect}}}\\ {I}_{j,2021}^{{\rm{indirect}}}=IN{F}_{j,2021}\ast {I}_{j,2015}^{{\rm{indirect}}}\\ {I}_{j,m,2022}^{{\rm{indirect}}}=CP{I}_{j,m,2022}\ast {I}_{j,2015}^{{\rm{indirect}}}\end{array}\right.$$

Note that ${I}_{j,2011}^{{\rm{indirect}}}$ and ${I}_{j,2015}^{{\rm{indirect}}}$ are generated from the 3EID dataset, which applied the 2011 and 2015 Japanese input-output tables, respectively. Equation (5) comprises three parts: 2012–2014, a simple linear interpolation method was applied; 2016–2021, modification factors that consider inflation (*INF*_*j,y*_), derived from the Economic and Social Research Institute, Cabinet Office of Japan, were used to modify the value of intensities; 2022, since the inflation information for the year was not available when this research was carried out, we referred to the 2020-Base CPI datasets, which are updated monthly by the Statistics of Japan, to obtain the monthly CPI data (*CPI*_*j,m*,2022_)^61^.

Another noteworthy improvement over previous studies was the emission intensity of electricity in different regions. The disparity in emissions across regional power grids is largely attributed to the dissimilarities in energy structures and consumption habits across distinct geographical areas^62,63^. Although some localities depend on coal-fired power generation, others adopt a greater proportion of clean energy resources. In addition, more advanced regions potentially exhibit greater reliance on high-tech industries, and less developed regions may depend more heavily on traditional industries, further augmenting differences in power grid emissions^64^.

Therefore, in this study, we referred to the ‘CO_2_ emission factors by electricity utility companies’ in the ‘Calculation Method and Emission Factors for Calculation, Reporting, and Disclosure System’ published by the Ministry of the Environment, Government of Japan^65^. This government report disclosed the CO_2_ emission factors of numerous electricity suppliers and provided corresponding calculation methods. To be more specific, we adopted the adjusted emission factor for calculation. This factor was adjusted by incorporating both domestic and international certified emission reductions, which provides a comprehensive and precise measure of the CO_2_ emissions generated during the electricity supply process across the various utilities under study.

Considering the structure of the electricity market in Japan, particularly the latest reforms, it is important to acknowledge that electricity retailers are not necessarily producers or distributors. However, by focusing on the 10 major electricity companies in Japan (Tokyo Electric Power Company, Kansai Electric Power Company, Chubu Electric Power Company, Hokkaido Electric Power Company, Chugoku Electric Power Company, Shikoku Electric Power Company, Kyushu Electric Power Company, Tohoku Electric Power Company, Hokuriku Electric Power Company, and Okinawa Electric Power Company) which collectively supply the majority of electricity consumers, this study was able to construct a representative picture of regional electricity consumption^66^. This, in turn, offers valuable insights into regional power generation patterns and the associated emissions. Therefore, this study used the emission factors of 10 major electricity companies to cover the emission factors of electricity in different regions of Japan to distinguish the variations between regions and cities.

Given all data preparations, the per capita indirect emissions ${E}_{i,j,m,y}^{{\rm{direct}}}$ can be obtained from household expenditures using Eq. (6).

6 $${E}_{i,j,m,y}^{{\rm{indirect}}}={c}_{i,j,m,y}\cdot {I}_{j,m,y}^{{\rm{indirect}}}/h{s}_{j,m,y}$$

## Data Records

This dataset contains the monthly per capita direct and indirect household carbon footprints for 51 cities, 10 regions, the national scale, large, medium, and small cities, and villages in Japan from 2011 to 2022. The data collected over 12 years have been uploaded to Figshare^67^ and can be organized into four categories: “Calculation results,” “Emission intensity,” “FIES,” and “Household size.” The “Calculation results” category contains the direct and indirect calculation results at both city and regional levels (the results for households nationwide and in different scales of cities are included in the results on the regional level). The “Emission intensity” category provides emission factors for four fuels (natural gas, gasoline, LPG, and kerosene) for gCO_2_/yen emitted. In contrast to previous studies that applied the same grid emission factor for all areas of Japan, this study combines region-specific carbon emission factors. These factors, measured in tons of CO_2_ per kWh, were provided by electricity utility companies and represent the indirect emission factors for electricity use in different regions. Detailed information is contained in a file called “indirect emission intensity.xlsx”. The “FIES” category contains three files, including a cross-mapping of consumption items in the 2015 FIES and 3EID databases (named Mapping.xlsx), details of pertinent industries in the FIES database (named FIES_items_Eng_2011-22.xlsx) and information on the classification method used in the result analysis (named Category.xlsx). Finally, the “Household size” category includes two files containing monthly household sizes for each region and city during the study period. Table 1 lists all the files, and Table 2 lists the records of the carbon footprint calculations for each year. This dataset contains 4,890,537 data points, including 4,852,845 indirect and 37,692 direct emissions.

**Table 1:** Summary of the dataset files and corresponding descriptions.

Filename	Description
CI_direct_2011.xlsx	*City-level direct emissions for 2011*
CI_direct_2012.xlsx	*City-level direct emissions for 2012*
…	*…*
CI_direct_2022.xlsx	*City-level direct emissions for 2022*
CI_indirect_2011.xlsx	*City-level indirect emissions for 2011*
CI_indirect_2012.xlsx	*City-level indirect emissions for 2012*
…	*…*
CI_indirect_2022.xlsx	*City-level indirect emissions for 2022*
RE_direct_2011.xlsx	*Regional-level direct emissions for 2011*
RE_direct_2012.xlsx	*Regional-level direct emissions for 2012*
…	*…*
RE_direct_2022.xlsx	*Regional-level direct emissions for 2022*
RE_indirect_2011.xlsx	*Regional-level indirect emissions for 2011*
RE_indirect_2012.xlsx	*Regional-level indirect emissions for 2012*
…	*…*
RE_indirect_2022.xlsx	*Regional-level indirect emissions for 2022*
CI_city_gas_intencity.xlsx	*City-level direct emission intensity for city gas from 2011 to 2022*
CI_gasoline_intensity.xlsx	*City-level direct emission intensity for gasoline from 2011 to 2022*
CI_kerosene_intensity.xlsx	*City-level direct emission intensity for kerosene from 2011 to 2022*
CI_lpg_intensity.xlsx	*City-level direct emission intensity for LPG from 2011 to 2022*
CI_city_electricity_intensity.xlsx	*City-level indirect emission intensity for electricity from 2011 to 2022*
RE_city_gas_intencity.xlsx	*Regional-level direct emission intensity for city gas from 2011 to 2022*
RE_gasoline_intensity.xlsx	*Regional-level direct emission intensity for city gas from 2011 to 2022*
RE_kerosene_intensity.xlsx	*Regional-level direct emission intensity for city gas from 2011 to 2022*
RE_lpg_intensity.xlsx	*Regional-level direct emission intensity for city gas from 2011 to 2022*
RE_regional_electricity_intensity.xlsx	*Regional-level direct emission intensity for city gas from 2011 to 2022*
Consumer Price Index.xlsx	*Data on CPI used to convert monthly indirect carbon emission intensities in 2022*
Category.xlsx	*Table comparing the distinct classification methodologies for items between this study and prior research*
FIES_items_Eng_2011-22.xlsx	*Data pertaining to the nomenclature of items within the FIES dataset*
Mapping.xlsx	*Comparison and correlation of data items between FIES and 3EID dataset*
City-Household size.xlsx	*Data regarding the number of individuals within a given household unit*
Region-Household size.xlsx	*Data regarding the number of individuals within a given household unit*

**Table 2:** Data records for each study year.

Year	National/city scales	Regions	Cities	Months	Direct emission		Indirect emission		Total data records
					Item number	Data records	Item number	Data records	
2011	5	10	51	12	4	3,168	515	407,880	411,048
2012	5	10	51	12	4	3,168	515	407,880	411,048
2013	5	10	52	12	4	3,216	515	414,060	417,276
2014	5	10	52	12	4	3,216	515	414,060	417,276
2015	5	10	52	12	4	3,216	515	414,060	417,276
2016	5	10	52	12	4	3,216	515	414,060	417,276
2017	5	10	52	12	4	3,216	515	414,060	417,276
2018	5	10	52	12	4	3,216	515	414,060	417,276
2019	5	10	52	12	4	3,216	515	414,060	417,276
2020	5	10	52	12	4	3,216	515	414,060	417,276
2021	5	10	52	12	4	3,216	515	414,060	417,276
2022	5	10	52	9	4	2,412	515	310,545	312,957

## Technical Validation

### Multiscale carbon footprint in Japan from 2011 to 2022

**Fig. 2:**
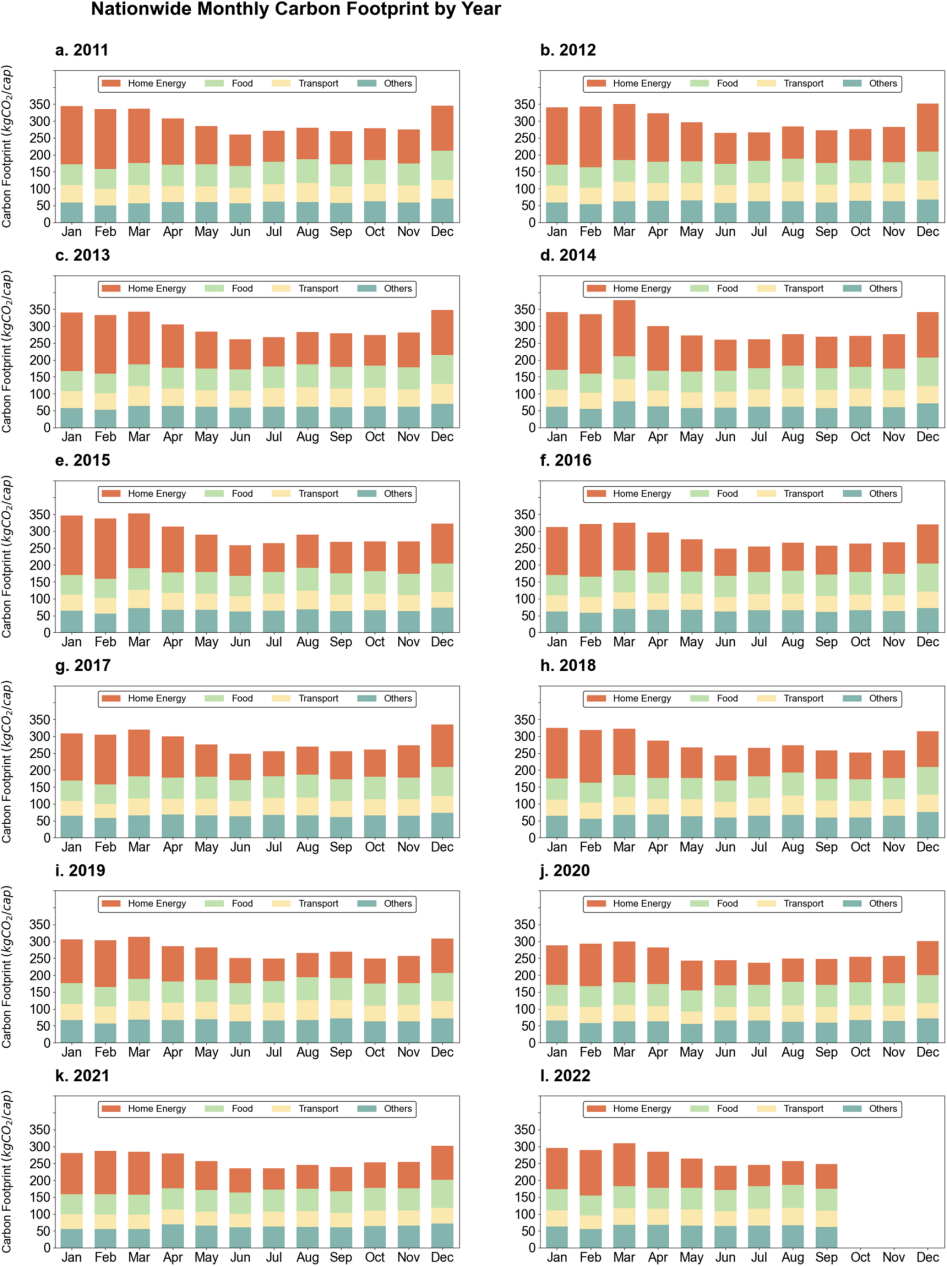
Japan’s monthly household carbon footprint from 2011–2022.

Fig. 2 shows the monthly carbon footprints of households across Japan from 2011 to September 2022. The results of the carbon footprint are divided into four major categories: ‘Household energy’,  ‘Food’, ‘Transport,’ and ‘Others, the details of which can be found in the file named Category.xlsx. In this study, direct and indirect household energy use are included in ‘Household energy,’ while gasoline combustion falls under the ‘Transport’ category. The ‘Food’ category considers only the carbon footprint of purchased ingredients and seasoning, excluding energy use involved in cooking. According to Fig. 2, the total carbon footprint of Japanese households is higher in winter, mainly driven by the demand for household energy. The average carbon footprints of the ‘Household energy’ and ‘Food’ categories are the highest, at 108.34 kg CO_2_/per capita/month and 65.58 kg CO_2_/per capita/month, respectively, while those of ‘Transport’ and ‘Others’ categories are lower. Furthermore, from 2013 to 2019, the carbon footprint of the Household energy category significantly decreased, while those of the ‘Others’ and ‘Food’ categories slightly increased.

**Fig. 3:**
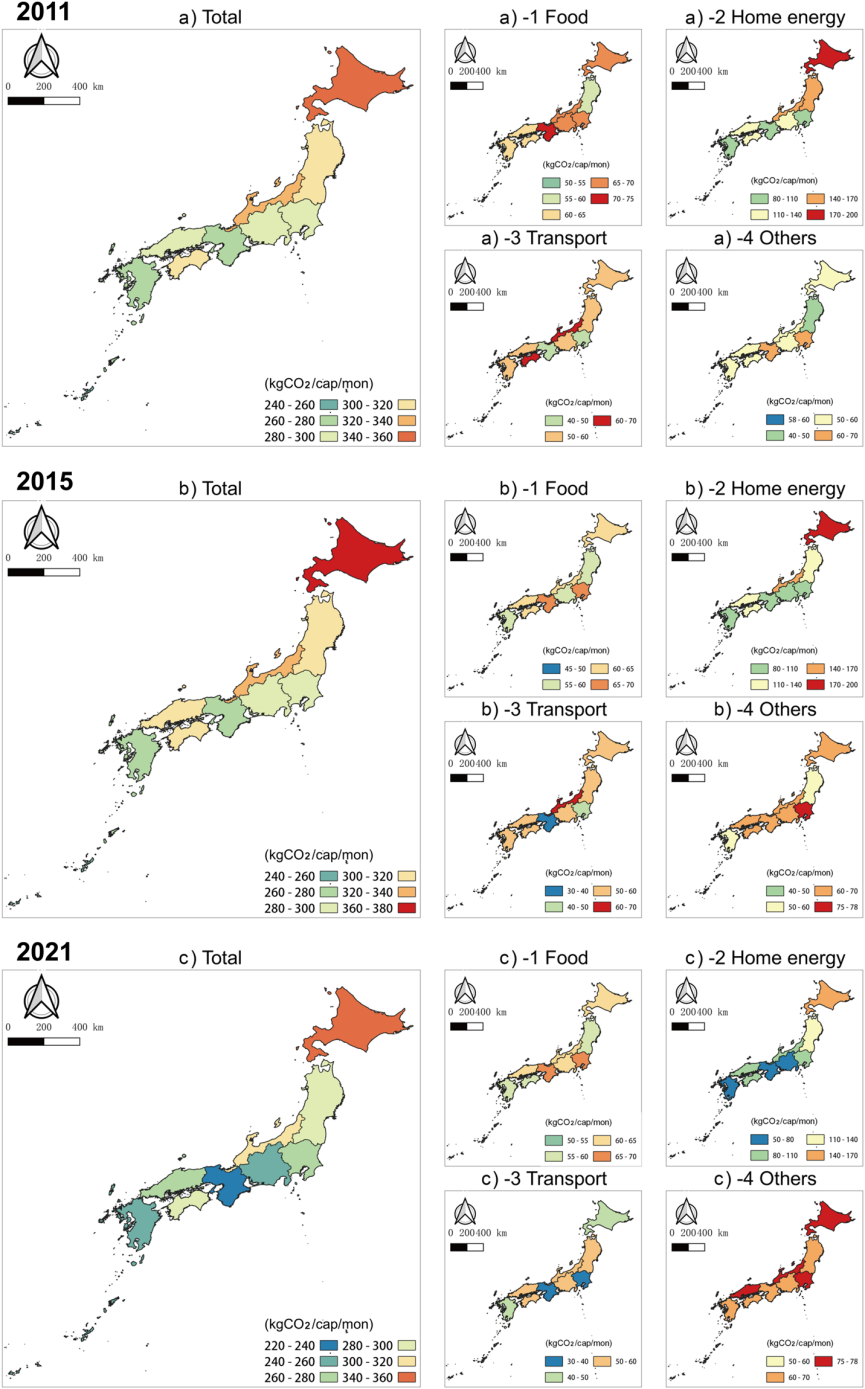
Per capita monthly carbon emissions and trends in 10 regions of Japan from 2011–2021.

In addition to the national level, this dataset also covers the household carbon footprint of 10 regions in Japan. For illustrative purposes, we’ve selected the results for three representative years. The per capita monthly carbon emissions across the 10 regions remained relatively stable from 2011 to 2015 (Fig. 3). However, there was a 5–15% reduction in emissions between 2015 and 2021. Emissions related to food showed a slight reduction from 2011 to 2015 but did not change significantly from 2015 to 2021. The trends in household energy-related emissions, which are the primary sources of carbon emissions, were consistent with the overall trend in carbon emissions, showing a significant reduction from 2015 to 2021. For example, the per capita monthly home energy-related carbon emissions in the Kinki region decreased by approximately 39 kg CO_2_/cap/mon, which is only 63% of the emissions recored in 2015. Emissions related to transportation showed an overall decreasing trend over the past decade, with a greater reduction from 2015 to 2021. The average reduction in emissions from 2011 to 2015 was approximately 5%, whereas that from 2015 to 2021 was approximately 10%. Carbon emissions from other sources steadily increased at a similar rate of approximately 12% during both periods.

**Fig. 4:**
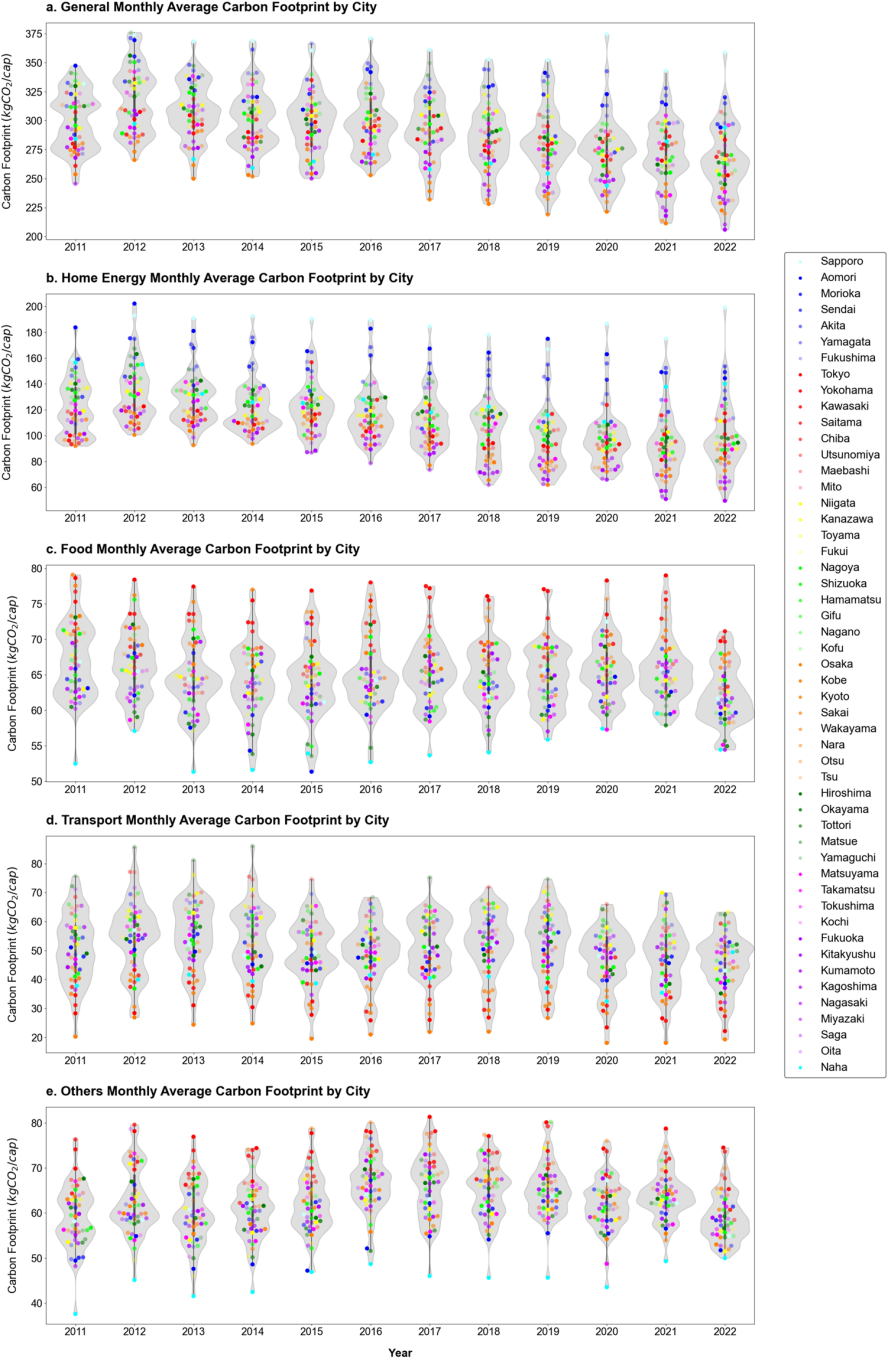
Violin map of monthly average household carbon footprint distribution in 51 Japanese cities from 2011 to 2022. Note: the data for 2022 does not cover all months of the year, as it only covers January to September.

The dataset’s final level comprises cities. Because of the large number of cities covered, we partially summarized the data and displayed the average results for all months in each year in Fig. 4. Overall, the total carbon footprints of northern cities, such as Sapporo, Aomori, and Sendai, are generally higher, whereas southern cities in the Kyushu and Kansai regions tend to have lower carbon footprints, as shown in Fig. 4a. Household carbon footprints fluctuated from 2011 to 2016 but generally decreased after 2016, with regional disparities remaining evident. The carbon footprint caused by home energy use (Fig. 4b) has been  on a gradual decline since 2012, though regional differences still exist. The carbon footprints caused by food consumption (Fig. 4c) vary among cities. Kyoto and Kawasaki have the highest carbon footprints, and Naha has the lowest, showing a stable trend with a relatively even distribution. The carbon footprint due to transportation exhibits a mild decrease overall (Fig. 4d), with emissions from transportation in major cities such as Tokyo, Yokohama, and Kyoto being relatively low. In contrast, emissions in some cities located in the central or eastern Honshu, western Japan, Shikoku, and Kyushu regions are higher, which are closely related to local public transportation systems. The carbon footprint of ‘Others’ (Fig. 4e) shows a fluctuating trend, with major cities in the Kanto region having higher carbon footprints in this category, while Naha and other less economically developed cities have lower carbon footprints.

### Comparison with other relevant databases

**Fig. 5:**
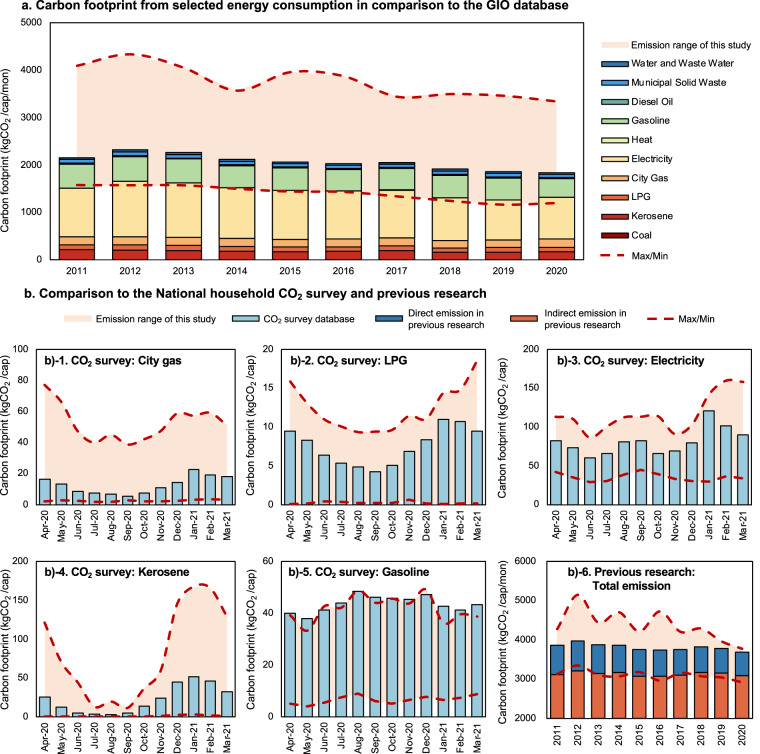
Results validation with GIO data, National Household CO_2_ Survey, and previous research. The carbon footprint from selected energy consumption compared to (**a**) the GIO database, (**b**–**b**)-**1**- **b)-5**: the National Household CO_2_ Survey, and **b)-6**: previous research.

**Fig. 6:**
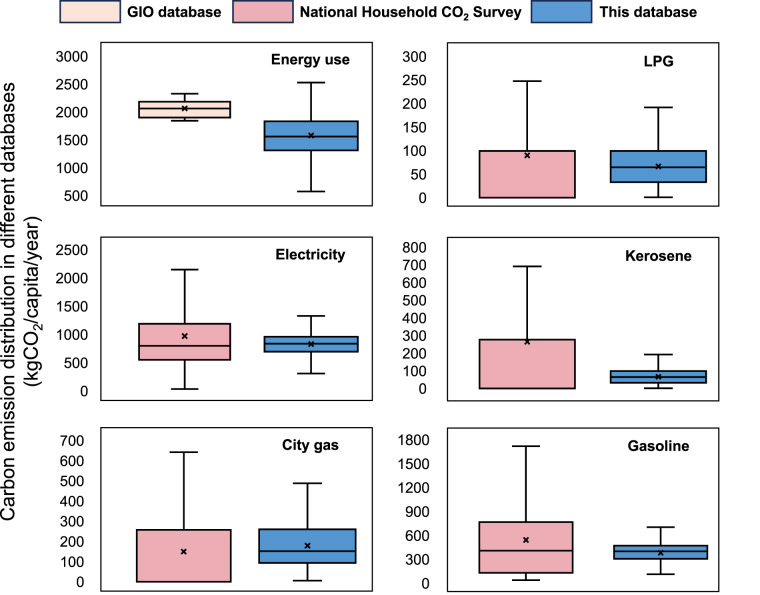
Carbon footprint distribution in GIO database, National Household CO_2_ Survey, and this study.

To validate the accuracy of our study, we consulted carbon footprint data from several government agencies, including the GHG Emissions Data of Japan from the Greenhouse Gas Inventory Office of Japan (GIO)^68^ at the National Institute for Environmental Studies (NIES), the National Household CO_2_ Survey from the Japan Society of Energy and Resources, and a previous study^54^. The detailed results of these comparisons by year and month are shown in Fig. 5, and the overall quantitative assessment results are summarized in Table 3 and Fig. 6.

**Table 3:** Quantitative assessment results of the comparison with other databases.

Average carbon footprint by category (kgCO_2_ per capita)	This dataset	GIO dataset	National Household CO_2_ Survey	Previous research
Energy-use (annual)	1579.80 (1551.08, 1608.51)	2062.53 (1948.00, 2177.10)	/	/
Total (annual)	3126.26	/	/	3819.17
City gas	15.09 (14.37,15.81)	14.13 (13.88, 14.38)	12.42 (11.99, 12.84)	17.34
LPG	5.49 (5.22, 5.77)	8.23 (7.93, 8.52)	7.48 (7.20, 7.77)	8.42
Electricity	69.57 (68.16, 70,97)	83.97 (78.31, 89.63)	80.86 (79.75, 81.96)	/
Kerosene	12.68(11.48, 13.87)	15.39 (14.39, 16.40)	22.05 (21.11,22.98)	11.87
Gasoline	28.82(28.09, 29.55)	39.07 (37.36, 40.77)	41.92 (41.03, 42.80)	16.51

Note: 95% confidence interval of the carbon footprint in each database is provided in (), and the average carbon footprints for specific fuels are monthly data.

Figure 5a displays a bar chart showing emissions by fuel type from GIO, including coal, kerosene, LPG, city gas, electricity, heat, gasoline, diesel oil, municipal solid waste, water, and wastewater. The y-axis represents emissions in kg CO_2_/capita, and the x-axis shows data from 2011 to 2020. The shaded areas represent the emission ranges used in this study. While our study classified indirect sources such as electricity, water, and wastewater emissions, we validated our calculation results by selecting indirect emission results of specific energy consumption categories, including ‘Electricity Bill for Late-Night Electricity,’ ‘Other Electricity Bills,’ and ‘Other Light Heat Other’; we found that the selected energy consumption by GIO corresponds with our calculation scope. We also compared our results to the National Household CO_2_ Survey in Reiwa 2 (from April 2020 to March 2021), with Fig. 5b)-1 to b)-5 displaying CO_2_ emissions for city gas, LPG, electricity, kerosene, and gasoline, respectively. Each corresponding month’s average per-capita emissions are displayed in each column to account for the large variations in emissions reported by over 10,000 households in the survey. Our results indicated that, except for gasoline, the survey data were consistent with our calculated results, and the emissions from gasoline were only slightly higher than our maximum value in some months. Figure 5b)-6 showcases a comparison of our results with those obtained in previous research.

Table 3 presents a quantitative assessment of these comparisons, illustrating the average annual emissions and 95% confidence intervals for the carbon footprints across various energy usage types. In addition, Fig. 6 offers a box plot representation of data distributions in the GIO database, the National Household CO_2_ Survey, and this database. Each box plot includes median, upper, and lower quartiles, encapsulating the data distribution within each database.

The notable consistency between these datasets lends further credence to our model, while any discrepancies prompt useful avenues for further exploration and refinement of our approach.

## Usage Notes

This dataset aggregates data from multiple sources, including the FIES and 3EID datasets, and some disclosure documents from the Ministry of the Environment of Japan. One of the main tasks in establishing this dataset is to cross-map the large FIES and 3EID databases to obtain consistent consumption categories. It should be noted that the consumption categories included in the household survey data published by the FIES underwent multiple changes over different time periods, which posed certain difficulties in the calculation. For example, since 2020, FIES has eliminated consumption categories such as “43X midnight low electricity rate” and “430 other electricity”. It now only provides consumption data for “3.1 electricity charges”. Therefore, appropriate measures were taken during data processing to ensure reliability and comparability.

Despite providing valuable information, we must also acknowledge the limitations of this dataset. First, for city-level quantification, the coverage of our dataset was limited to prefectural-level cities, excluding Japan’s medium-sized cities and rural areas. This limitation may result in incomplete or inaccurate carbon footprint data for certain regions, affecting the overall analysis. Second, Japan is currently facing population concentration in large cities (especially among the younger generation) and an aging population, which may also have led to the incomplete and unrepresentative data obtained in this study. Future research should include a wider range of regions to fully understand the impact of the household carbon footprint on climate change and provide more accurate and comprehensive data to support effective environmental policies.

## Data Availability

The code used for analysis in this study is publicly available at https://github.com/LiqiaoHuang/Household-carbon-footprint-quantification.git.

## References

[1] Liu ZM, Espinosa P (2019). Tackling climate change to accelerate sustainable development. Nature Climate Change.

[2] Mikhaylov A, Moiseev N, Aleshin K, Burkhardt T (2020). Global climate change and greenhouse effect. Entrepreneurship and Sustainability Issues.

[3] Kweku DW (2018). Greenhouse effect: greenhouse gases and their impact on global warming. Journal of Scientific research and reports.

[4] Mallapaty S (2020). How china could be carbon neutral by mid-century. Nature.

[5] Salvia, M. *et al*. Will climate mitigation ambitions lead to carbon neutrality? An analysis of the local-level plans of 327 cities in the EU. *Renewable & Sustainable Energy Reviews* **135**, 10.1016/j.rser.2020.110253 (2021).

[6] Dahal K, Niemela J (2016). Initiatives towards Carbon Neutrality in the Helsinki Metropolitan Area. Climate.

[7] C40. Legal interventions: How cities can drive climate action. (2021).

[8] Nations, U. Vol. pp. 1-27. (United Nations, Paris, 2015).

[9] United Nations Development Programme, U. *Net Zero Pathways*, https://climatepromise.undp.org/what-we-do/areas-of-work/net-zero-pathways (2023).

[10] Wang, F. *et al*. Technologies and perspectives for achieving carbon neutrality. *Innovation* **2**, 10.1016/j.xinn.2021.100180 (2021). 10.1016/j.xinn.2021.100180 PMC8633420 34877561

[11] Fajardy M, Mac Dowell N (2017). Can BECCS deliver sustainable and resource efficient negative emissions?. Energy & Environmental Science.

[12] Bataille C (2018). A review of technology and policy deep decarbonization pathway options for making energy-intensive industry production consistent with the Paris Agreement. Journal of Cleaner Production.

[13] UN. 17 Sustainable Development Goals. (2015).

[14] Long Y, Yoshida Y (2018). Quantifying city-scale emission responsibility based on input-output analysis–Insight from Tokyo, Japan. Applied energy.

[15] Marcotullio PJ (2014). Urbanization and the carbon cycle: Contributions from social science. Earths Future.

[16] Bachmann C, Roorda MJ, Kennedy C (2015). Developing a multi-scale multi-region input-output model. Economic Systems Research.

[17] Toufani, P., Kucukvar, M., Onat, N. C. & Ieee. in *IEEE International Conference on Industrial Engineering and Engineering Management (IEEE IEEM)*. 11–16 (2018).

[18] Shi, S. Q. & Yin, J. H. Global research on carbon footprint: A scientometric review. *Environmental Impact Assessment Review* **89**, 10.1016/j.eiar.2021.106571 (2021).

[19] Long Y, Yoshida Y, Zhang R, Sun L, Dou Y (2018). Policy implications from revealing consumption-based carbon footprint of major economic sectors in Japan. Energy Policy.

[20] Nansai K (2009). Improving the Completeness of Product Carbon Footprints Using a Global Link Input–Output Model: The Case of Japan. Economic Systems Research.

[21] Yang Y, Ingwersen WW, Hawkins TR, Srocka M, Meyer DE (2017). USEEIO: A new and transparent United States environmentally-extended input-output model. Journal of Cleaner Production.

[22] Feng K, Davis SJ, Sun L, Hubacek K (2015). Drivers of the US CO 2 emissions 1997–2013. Nature communications.

[23] Paladugula AL (2018). A multi-model assessment of energy and emissions for India’s transportation sector through 2050. Energy Policy.

[24] Lee J, Taherzadeh O, Kanemoto K (2021). The scale and drivers of carbon footprints in households, cities and regions across India. Global Environmental Change.

[25] Mi ZF (2016). Consumption-based emission accounting for Chinese cities. Applied Energy.

[26] Jiang YD, Long Y, Liu QL, Dowaki K, Ihara T (2020). Carbon emission quantification and decarbonization policy exploration for the household sector - Evidence from 51 Japanese cities. Energy Policy.

[27] Long, Y. *et al*. Comparison of city-level carbon footprint evaluation by applying single- and multi-regional input-output tables. *Journal of Environmental Management* **260**, 10.1016/j.jenvman.2020.110108 (2020). 10.1016/j.jenvman.2020.110108 32090821

[28] Cortekar J, Bender S, Brune M, Groth M (2016). Why climate change adaptation in cities needs customised and flexible climate services. Climate Services.

[29] Heiskanen E, Jalas M, Rinkinen J, Tainio P (2015). The local community as a “low-carbon lab”: Promises and perils. Environmental Innovation and Societal Transitions.

[30] Pulselli, R. M. *et al*. Future city visions. The energy transition towards carbon-neutrality: lessons learned from the case of Roeselare, Belgium. *Renewable & Sustainable Energy Reviews* **137**, 10.1016/j.rser.2020.110612 (2021)

[31] Fragkias M, Lobo J, Strumsky D, Seto KC (2013). Does Size Matter? Scaling of CO2 Emissions and US Urban Areas. Plos One.

[32] Wiedmann TO, Chen GW, Barrett J (2016). The Concept of City Carbon Maps: A Case Study of Melbourne, Australia. Journal of Industrial Ecology.

[33] Sturiale L, Scuderi A (2019). The role of green infrastructures in urban planning for climate change adaptation. Climate.

[34] Vaidya, H. & Chatterji, T. SDG 11 Sustainable Cities and Communities SDG 11 and the New Urban Agenda: Global Sustainability Frameworks for Local Action. *Actioning the Global Goals for Local Impact: Towards Sustainability Science, Policy, Education and Practice*, 173–185, 10.1007/978-981-32-9927-6_12 (2020).

[35] Roy, J., Some, S., Das, N. & Pathak, M. Demand side climate change mitigation actions and SDGs: literature review with systematic evidence search. *Environmental Research Letters* **16**, 10.1088/1748-9326/abd81a (2021).

[36] Maizlish N (2013). Health Cobenefits and Transportation-Related Reductions in Greenhouse Gas Emissions in the San Francisco Bay Area. American Journal of Public Health.

[37] Long, Y. *et al*. PM2.5 and ozone pollution-related health challenges in Japan with regards to climate change. *Global Environmental Change-Human and Policy Dimensions* **79**, 10.1016/j.gloenvcha.2023.102640 (2023).

[38] Andrade JCS, Dameno A, Perez J, Almeida JMD, Lumbreras J (2018). Implementing city-level carbon accounting: A comparison between Madrid and London. Journal of Cleaner Production.

[39] Long Y, Yoshida Y (2018). Quantifying city-scale emission responsibility based on input-output analysis - Insight from Tokyo, Japan. Applied Energy.

[40] Kanemoto K, Shigetomi Y, Hoang NT, Okuoka K, Moran D (2020). Spatial variation in household consumption-based carbon emission inventories for 1200 Japanese cities. Environmental Research Letters.

[41] Long Y (2021). Monthly direct and indirect greenhouse gases emissions from household consumption in the major Japanese cities. Scientific Data.

[42] Zheng SQ, Wang R, Glaeser EL, Kahn ME (2011). The greenness of China: household carbon dioxide emissions and urban development. Journal of Economic Geography.

[43] Li JS (2018). Carbon emissions and their drivers for a typical urban economy from multiple perspectives: A case analysis for Beijing city. Applied Energy.

[44] Wiedenhofer D (2017). Unequal household carbon footprints in China. Nature Climate Change.

[45] Sudmant A, Gouldson A, Millward-Hopkins J, Scott K, Barrett J (2018). Producer cities and consumer cities: Using production- and consumption-based carbon accounts to guide climate action in China, the UK, and the US. Journal of Cleaner Production.

[46] Harris, S., Weinzettel, J., Bigano, A. & Kallmen, A. Low carbon cities in 2050? GHG emissions of European cities using production-based and consumption-based emission accounting methods. *Journal of Cleaner Production* **248**, 10.1016/j.jclepro.2019.119206 (2020)

[47] Ivanova D (2020). Quantifying the potential for climate change mitigation of consumption options. Environmental Research Letters.

[48] Dietz T, Gardner GT, Gilligan J, Stern PC, Vandenbergh MP (2009). Household actions can provide a behavioral wedge to rapidly reduce US carbon emissions. Proceedings of the National Academy of Sciences.

[49] Foteinis, S. How small daily choices play a huge role in climate change: The disposable paper cup environmental bane. *Journal of Cleaner Production* **255**, 10.1016/j.jclepro.2020.120294 (2020).

[50] Berners-Lee M, Hoolohan C, Cammack H, Hewitt CN (2012). The relative greenhouse gas impacts of realistic dietary choices. Energy Policy.

[51] Reichert A, Holz-Rau C, Scheiner J (2016). GHG emissions in daily travel and long-distance travel in Germany - Social and spatial correlates. Transportation Research Part D-Transport and Environment.

[52] Koide R (2021). Exploring carbon footprint reduction pathways through urban lifestyle changes: a practical approach applied to Japanese cities. Environmental Research Letters.

[53] Communications, J. M. O. I. A. A. (ed Statistics Bureau of Japan) (2023).

[54] Long Y (2023). Japanese urban household carbon footprints during early-stage COVID-19 pandemic were consistent with those over the past decade. npj Urban Sustainability.

[55] ANRE, A. F. N. R. A. E. (2020).

[56] Agency for Natural Resources and Energy, J. Oil product price. https://www.enecho.meti.go.jp/statistics/petroleum_and_lpgas/pl007/results.html

[57] Agency of Natural Resource and Energy, J. Japan Energy White Book 2018 (2018).

[58] Oil Information Center, t. I. o. E. E., Japan LPG consumption survey of Japan. https://oil-info.ieej.or.jp/documents/data/20080303_2.pdf (2006).

[59] Nansai, K., Moriguchi, Y., Tohno, S. *Embodied Energy and Emission Intensity Data for Japan Using Input-Output Tables*. (2002).

[60] Long Y, Yoshida Y, Fang K, Zhang HR, Dhondt M (2019). City-level household carbon footprint from purchaser point of view by a modified input-output model. Applied Energy.

[61] Statistics, J. G. Consumer Price Index. https://www.e-stat.go.jp/stat-search/files?page=1&layout=datalist&toukei=00200573&tstat=000001044944&cycle=7&year=20150&tclass1=000001044990&cycle_facet=cycle (2023).

[62] Gonocruz, R. A., Uchiyama, S. & Yoshida, Y. Modeling of large-scale integration of agrivoltaic systems: Impact on the Japanese power grid. *Journal of Cleaner Production* **363**, 10.1016/j.jclepro.2022.132545 (2022).

[63] Wei, W. D. *et al*. Multi-scope electricity-related carbon emissions accounting: A case study of Shanghai. *Journal of Cleaner Production* **252**, 10.1016/j.jclepro.2019.119789 (2020).

[64] Xie R, Fang JY, Liu CJ (2017). The effects of transportation infrastructure on urban carbon emissions. Applied Energy.

[65] Ministry of Environment, J. in https://ghg-santeikohyo.env.go.jp/calc (2023).

[66] The Federation of Electric Power Companies of Japan, F. *Ten Electric Power Companies as Responsible Suppliers of Electricity*, https://www.fepc.or.jp/english/energy_electricity/company_structure/index.html (2023).

[67] Long Y (2023). Figshare.

[68] Ministry of the Environment, J. & Greenhouse Gas Inventory Office of Japan (GIO), C., NIES (2022).

