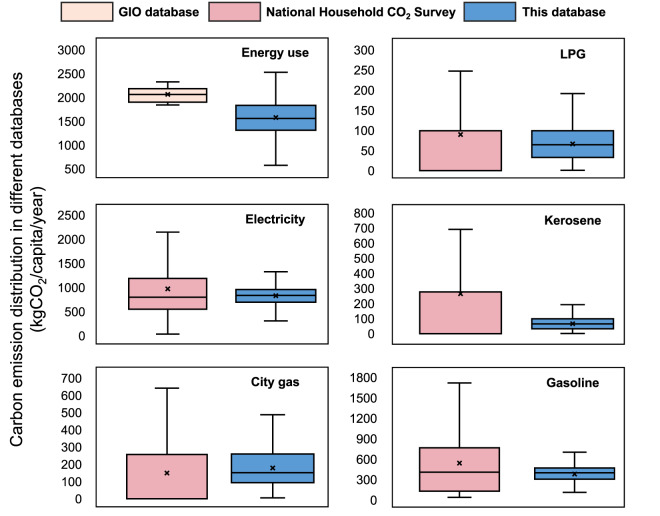# Publisher Correction: Extension and update of multiscale monthly household carbon footprint in Japan from 2011 to 2022

**DOI:** 10.1038/s41597-023-02381-y

**Published:** 2023-07-18

**Authors:** Liqiao Huang, Sebastian Montagna, Yi Wu, Zhiheng Chen, Kenji Tanaka, Yoshikuni Yoshida, Yin Long

**Affiliations:** 1 Graduate School of Engineering, University of Tokyo, Tokyo, Japan; 2 Bartlett School of Sustainable Construction, University College London, London, WC1E 7HB UK

**Keywords:** Environmental impact, Psychology and behaviour, Environmental impact, Psychology and behaviour

Correction to: *Scientific Data* 10.1038/s41597-023-02329-2, published online 08 July 2023

In this article the wrong figure appeared as Fig. 6; the figure should have appeared as shown below. The original article has been corrected.